# Data on the effects of fertilization on growth rates, biomass allocation, carbohydrates and nutrients of nitrogen-fixing and non-nitrogen-fixing tree legumes during tropical forest restoration

**DOI:** 10.1186/s13104-021-05552-5

**Published:** 2021-04-21

**Authors:** Roberto K. Jaquetti, José Francisco C. Gonçalves

**Affiliations:** Laboratory of Plant Physiology and Biochemistry, National Institute for Amazonian Research-INPA, Ave. André Araújo, 2936, Aleixo, Manaus, AM 69011-970 Brazil

**Keywords:** Amazon basin, Biomass growth, Carbon stocks, Degraded areas, Ecological restoration, Fabaceae, N-fixers, Plant nutrition, Soluble sugars, Starch

## Abstract

**Objectives:**

Tree legume species play an important role in forest restoration in the tropics. Understanding how different species adjust carbohydrate allocation and growth under distinct nutrient availability will enhance the success of restoring degraded areas.

**Data description:**

A 2-year tropical forest plantation of the Forest Restoration Program of the Balbina Hydropower Dam was evaluated. Three non-N-fixing (*Cenostigma tocantinum*,* Dipteryx odorata* and *Senna reticulata*) and three N-fixing (*Clitoria fairchildiana*, *Inga edulis* and *Acacia* spp.) tree legume species were either fertilized or not fertilized. Growth rates and biomass allocation were calculated, and carbon (C) fractions and nitrogen (N), phosphorus (P) and nonstructural carbohydrate (NSC) concentrations were determined. Multiple nutrient additions increased the growth rates and aboveground biomass production of fertilized plants. According to the results presented, different species and N- fixers respond differently to fertilization regimes. The authors encourage the use of the presented data in meta-analysis studies that consider the fertilization or nutrient deficiency effects on growth, carbohydrate and nutrient responses. N-fixing species with high biomass growth and foliar N are important for restoring N and C cycles in nutrient-limited soils. Fertilization treatments are fundamental during the early stages of forest plantation development.

## Objective

The present dataset aims to describe how Amazonian tree legumes and *Acacia* adjust growth and carbohydrate allocation under nutrient deficiencies and fertilization. The dataset of fertilized and unfertilized plants may be useful for practitioners during forest restoration and policymakers planning greenhouse gas mitigation efforts. Meta-analysis and global vegetation modeling studies on growth and carbohydrate allocation adjustments to fertilization treatments should consider the use of the present data. The study and use of N- fixers may assist in turning degraded areas into productive systems in communities that do not have access to mineral fertilizers. The data for the growth, carbohydrate and nutrient variables were used in the following publications:

Jaquetti RK. Ecophysiology of Fabaceae tree species during forest restoration in the Balbina Hydroeletric Dam in Amazonas State. Thesis, National Institute for Amazonian Research. 2018.

Pereira LOV. Dinâmica de nitrogênio no contínuo solo-planta em área alterada e cultivada com Fabaceae arbóreas submetidas à fertilização mineral. Dissertation, National Institute for Amazonian Research. 2017.

Jaquetti RK, Nascimento HEN, Zotarelli L, Rathinasabapathi B, Gonçalves JFC. Coordinated adjustments of carbohydrates and growth of tree legumes under different fertilization regimes in degraded areas in Amazonia. New Forests 2021, under minor revision.

Jaquetti RK, Gonçalves JFC, Nascimento HEM, da Costa KCP, Maia JMF, Schimpl FC. Fertilization and seasonality influence on the photochemical performance of leguminous tree species during Amazonian reforestation. PLoS One 2021, under minor revision.

## Data description

### Experiment and methods

The experiment consisted of two fertilization regimes of unfertilized and fertilized treatments. The 12 treatments were a combination of six species and two fertilization treatments. All treatments were planted in the nine blocks (n = 9). Due to mortality repetitions were lost in some treatments. A description of the experiment, including the species used and fertilization treatments, can be found in [1].

### Data collection and results

#### Precipitation

Mean monthly precipitation was measured at the Balbina Hydropower Dam climatic stationAM, Brazil. As presented in Figure 1, the 2015/16 El Niño Southern Oscillation caused a 60-day period with precipitation close to zero (Data file 1).

#### Soil chemical analyses

Eight soil samples were collected prior to fertilization in the study area at two depths, 0–15 and 15–30 cm (Data set 1). Soil organic matter and N, P, potassium (K), calcium (Ca), magnesium (Mg), iron, zinc and copper concentrations are presented in Table 1 (Data file 2). Mehlich-1 extracted P was determined by spectrophotometry, K by flame photometry, and Ca, Mg and micronutrients by spectrophotometry [2].

#### Growth analysis and C stocks

The biomass allocation was calculated as described by [3], and the absolute and relative growth rates in diameter, height and biomass were calculated according to [4]. The annual increase in C stocks (∆C_B_) was estimated for each individual tree as the product of the biomass production and C fractions [5].

Table 2 shows the significant differences for fertilization and species effects in growth variables (Data file 2). Nutrient additions enhanced the growth of fertilized plants (Figure 2 and Table 3) (Data files 1 and 2). N-fixing species showed increased biomass growth while unfertilized plants showed increased root mass fraction (RMF) (Figure 3) (Data file 1). The N-fixing *Acacia* had mean ∆C_B_ values of 26.5 kg C year^−1^ (Table 4) (Data file 2).

#### Nutrients and nonstructural carbohydrates

Oven-dried samples of leaf, stem and root material were used for nutrient and carbohydrate analysis. The N concentrations and C fractions were assessed with a 2400 Series II CHNS/O Organic Elemental Analyzer (PerkinElmer Inc., Waltham, MA, USA). P concentrations were determined by spectrophotometry [2]. Soluble sugars and starch were determined by the colorimetric method [6].

Statistical differences were found among species for carbohydrate and nutrient variables (Data file 2). N-fixing species presented higher N concentrations in leaves (N_L_) (Table 5) (Data file 2). The highest leaf-to-root starch ratio (starch_L_:starch_R_) values were found in the fertilized *Acacia* (Table 6) (Data file 2).

#### Correlations

Figure 4 presents positive relationships between the RMF and starch concentration in roots (starch_R_) (R^2^ = 0.37) (Eq. 1) and between ∆C_B_ and RGR_D_ (R^2^ = 0.92) (Eq. 2) (Data file 1).

1 $${\text{RMF}} = 0.014 \cdot starch_{R}^{0.49}$$

2 $$\Delta {\text{C}}_{B} = 0.11 \cdot RGR_{D}^{7.38}$$

### Data analysis

Two-way ANOVA was performed to assess the differences between fertilization treatments and among species. Means were compared using Tukey’s test, with a level of probability of *P* < 0.05. Statistical analyses were performed using STATISTICA 12.0 (StatSoft Inc., Tulsa, OK, USA). The raw data used and main results are summarized in dataset and data files described in Table 1.

**Table 1:** Overview of data files/data sets

Label	Name of data file/data set	File type (file extension)	Data repository and identifier (DOI or accession number)
Data file 1	Figures Precipitation_RGR diameter_correlation	PDF file (.pdf)	Figshare: https://doi.org/10.6084/m9.figshare.12869960.v6 [8]
Data file 2	Tables Growth rates_nutrients_carbon stock_carbohydrates_soil chemical	PDF file (.pdf)	Figshare: https://doi.org/10.6084/m9.figshare.12869960.v6 [8]
Dataset 1	Data availability Jaquetti and Gonçalves 2021	MS Excel file (.xlsx)	Figshare: https://doi.org/10.6084/m9.figshare.12869960.v6 [8]

## Limitations

- Data collection was performed in a single 3 ha area of the Forest Restoration Program of the Balbina Hydropower Dam; therefore, the fertilization and nutrient deficiency responses are comparable under similar edaphic and climatic characteristics.
- Great variation within laboratories and methods for carbohydrate concentration measurements makes it difficult to compare data [7]. However, using standard methods enables the detection of fertilization and species effects within one laboratory.

## Data Availability

The data described in this Data note can be freely and openly accessed on: [data files] Jaquetti RK. Growth, biomass allocation, carbohydrate concentrations, nutrient concentrations, soil data and precipitation data Jaquetti and Gonçalves 2021. Figshare. 2020. https://doi.org/10.6084/m9.figshare.12869960.v6. Please see Table 1 and reference [8] for details and links to the data.

## Abbreviations

C: Carbon
N: Nitrogen
P: Phosphorus
K: Potassium
Ca: Calcium
Mg: Magnesium
AGR_Bio_: Absolute growth rate in biomass
∆C_B_: Annual increase in biomass C stocks
∆C_G_: Annual increase in C stocks due to biomass growth
RMF: Root mass fraction
starch_R_: Starch concentration in root
